# Association of acute-phase plasma pTau217 levels with long-term post-stroke cognitive impairment

**DOI:** 10.3389/fnagi.2026.1839662

**Published:** 2026-07-23

**Authors:** Mónica Macías, Elena Escriche, María Molina, Inhar Esnaola, Adrián Jiménez, Sara Razquin-Sola, Miren Roldan, Amaya Urdánoz-Casado, Nuria Sobrino, Celia Fernández, Idoia Rubio, Dagoberto Aspra, Carlota Jáuregui, Roberto Muñoz, Maria Herrera, Maite Mendioroz

**Affiliations:** 1 Neuroepigenetics Unit-Navarrabiomed, University Hospital of Navarra, Universidad Pública de Navarra, Navarra Institute for Health Research (IdiSNA), Pamplona, Spain; 2 Redes de Investigación Cooperativa Orientadas a Resultados en Salud (RICORS)- ISCIII, Madrid, Spain; 3 Neurology Department, University Hospital of Navarra, Pamplona, Spain; 4 Radiology Department, University Hospital of Navarra, Pamplona, Spain; 5 Neurology Department, University Hospital of Navarra, Navarra Institute for Health Research (IdiSNA), Pamplona, Spain

**Keywords:** biomarker, neurodegeneration, post-stroke cognitive impairment (PSCI), pTau217, stroke

## Abstract

**Background:**

Post-stroke cognitive impairment (PSCI) is a common complication affecting stroke survivors, yet early biomarkers for risk stratification remain scarce. Plasma pTau217 has emerged as a specific biomarker for Alzheimer’s disease pathology. This study explores whether acute-phase pTau217 levels are associated with PSCI.

**Methods:**

We conducted a nested case-control study including 48 patients who developed PSCI within 5 years and 48 age and sex-matched patients as controls without cognitive decline. Plasma pTau217 was measured within 24 h after stroke onset using SIMOA. Statistical analysis included multivariable logistic regression, dose-response assessment via quartile stratification, and ROC curves.

**Results:**

Plasma pTau217 levels were significantly higher in PSCI cases than controls [0.313 (0.196–0.562) vs. 0.203 (0.126–0.343) pg/mL; *p-*value < 0.01]. Notably, pTau217 levels were not correlated with stroke severity (NIHSS at baseline) or atrial fibrillation, suggesting that pTau217 may reflect baseline neurobiological vulnerability. In multivariable models, ln-pTau217 levels were independently associated with PSCI risk (OR = 2.28; 95%CI = 1.19–4.37; *p*-value < 0.05). A significant linear dose-response relationship was observed, with PSCI risk increasing from 29.2% in the lowest quartile to 64.0% in the highest (*p-*value for trend < 0.05). The biomarker alone showed a significant discriminative capacity for clinically documented PSCI (AUC = 0.660; 95%CI = 0.552–0.768; *p*-value < 0.01). Furthermore, sex-stratified analysis revealed that the association was primarily driven by females.

**Conclusion:**

Higher acute-phase pTau217 levels were associated with an increased likelihood of clinically documented cognitive impairment during follow-up. These exploratory findings suggest that acute-phase pTau217 may be associated with long-term cognitive outcomes after stroke, although larger prospective studies, integrating additional biomarkers with standardized neuropsychological assessment are needed.

## 1 Introduction

Stroke is a leading cause of disability worldwide, often facing long-term complications that significantly affect quality of life (GBD 2019 Stroke Collaborators, 2021). Among these complications, post-stroke cognitive impairment (PSCI) is one of the most debilitating, affecting up to 40%–60% of stroke survivors within the first year (El Husseini et al., 2023; Sexton et al., 2019). PSCI encompasses a spectrum of cognitive deficits, ranging from mild cognitive impairment to dementia, and is associated with increased morbidity, mortality, and healthcare costs. Despite its prevalence, early identification of patients at risk for PSCI remains challenging, due to limited sensitivity and specificity of current diagnostic tools (Godefroy et al., 2025a).

The pathophysiology of PSCI is multifactorial, involving a complex interplay of vascular injury, neurodegenerative processes, and neuroinflammation (Rost et al., 2022). Emerging evidence suggests that biomarkers of neurodegeneration may be useful for PSCI (Rost et al., 2021). Among these biomarkers, plasma phosphorylated tau (pTau) proteins have garnered significant attention due to their established role in Alzheimer’s disease (AD) and other tauopathies (Rawat et al., 2022; Xia et al., 2021). In particular, pTau217, a specific isoform of pTau, has shown promise as a highly sensitive and specific biomarker for AD, with recent studies demonstrating its ability to differentiate AD from other neurodegenerative conditions (Benussi et al., 2025; Palmqvist et al., 2020; Yu et al., 2023). The potential utility of pTau217 in stroke-related cognitive impairment, however, remains unexplored. Beyond its role in AD, pTau217 could identify a state of neurobiological vulnerability, where pre- existing neurodegenerative changes increase the susceptibility to cognitive decline after a vascular insult.

Ischemic stroke triggers a cascade of molecular events, including tau hyperphosphorylation and aggregation, which may contribute to the development of PSCI (Marklund et al., 2021). In addition, ischemic stroke and AD share other pathophysiological changes, including hypoperfusion, oxidative stress, neuroinflammation or vascular damage (Dong et al., 2018). Given the shared mechanisms between stroke-induced neurodegeneration and AD, pTau217 could serve as a valuable biomarker for for identifying individuals with underlying mixed vascular-neurodegenerative pathology who are at higher risk for PSCI development (Cui et al., 2018). This study aims to determine whether plasma pTau217 levels, measured using SIMOA technology (Rissin et al., 2010) during the acute phase of ischemic stroke, can identify patients with a pre-existing vulnerability to the development of PSCI.

## 2 Material and methods

We conducted a nested case-control study within a prospective group of patients diagnosed with acute ischemic stroke at University Hospital Navarra between November 2015 and December 2017. Patients were eligible for inclusion if they met the following criteria: (1) diagnosis of acute ischemic injury confirmed by diffusion-weighted MRI and (2) age ≥ 18 years. Informed consent was obtained from all participants, and the local Ethics Committee approved the study.

A total of 274 patients with acute ischemic stroke were screened for inclusion in the study. Within this group, incident post-stroke cognitive impairment (PSCI) cases were defined as ischemic stroke patients with documented cognitive dysfunction in the medical record at 5-year follow-up. Exclusion criteria included previous cognitive impairment, neurodegenerative disease, or severe psychiatric disorders. Non-PSCI controls were defined as ischemic stroke patients who were cognitively unimpaired at 5-year follow-up. Individuals with moderate-severe aphasia and severe disability (Modified Rankin Scale, mRS = 5) at discharge were also excluded to avoid bias in PSCI detection.

Baseline demographic and clinical characteristics were collected for all participants, including age, sex, a detailed history of vascular risk factors and comorbidities. To identify ischemic stroke etiology, electrocardiography (EKG), vascular imaging study (carotid ultrasonography or angio-TC), complete blood count and leucocyte differential and blood biochemistry, coagulation test, and Holter monitoring were performed in all patients. Additional studies such as transthoracic echocardiography or prolonged EKG monitoring were performed if necessary. Brain magnetic resonance imaging (MRI) was performed in all patients and findings were recorded. Stroke severity was assessed by using the National Institutes of Health Stroke Scale (NIHSS; NIH, 2025), and etiology was determined by the Trial of Org 10172 in Acute Stroke Treatment (TOAST; Adams et al., 1993) and Oxfordshire criteria (Bamford et al., 1991). EDTA plasma samples were collected by venous puncture from all participants within 24 h of stroke onset. Plasma was separated by centrifugation at 2,400 *g* for 15 min at room temperature and stored at −80 °C until analysis. Plasma pTau217 levels were quantified using the Simoa^®^ ALZpath p-Tau-217 Advantage PLUS kit (Catalog number: 104570) on a Simoa SR-X platform (Quanterix, Billerica, MA, USA). The assay was performed according to the manufacturer’s instructions, with a lower limit of detection (LOD) of 0.0004 pg/mL and a functional limit of quantification (LOQ) of 0.0129 pg/mL.

Two provided controls within the kit and an additional pool of samples were used as internal controls to ensure that the intra- and inter-assay coefficients of variation (CVs) remained below 20% across plates. To guarantee samples results accuracy, all samples were analyzed in duplicate, with PSCI cases and non-PSCI controls run within each plate. Only replicates with a CV ≤ 20% were considered for further statistical analysis.

Descriptive statistics were used to summarize demographic and clinical characteristics of patients. Depending on their distribution, continuous variables were expressed as mean ± standard deviation or median (interquartile range) and categorical variables were expressed as frequencies (percentages). A bivariate analysis was performed between PSCI cases and non-PSCI controls using Student’s *t*-test or Mann-Whitney U test for continuous variables and chi-square or Fisher’s exact test for categorical variables, as appropriate. Correlations were performed using Pearson/Spearman tests. Due to its right-skewed distribution, plasma pTau217 levels were natural log-transformed (ln) for multivariable logistic regression analyses to improve model stability and reduce the influence of extreme values. The association between plasma ln-pTau217 levels and PSCI was assessed using a backward multivariable logistic regression model, with PSCI as the dependent variable, pTau217 levels as the independent variable and including variables significantly different in the bivariate analysis. The internal robustness of the log-transformed model was further evaluated using bootstrap resampling with 1,000 iterations. A quartile-based stratification of pTau217 levels was conducted to test the absolute risk for PSCI development. Finally, the predictive accuracy of pTau217 and various multivariable logistic regression models for PSCI was evaluated using receiver operating characteristic (ROC) curve analysis, with the area under the curve (AUC) calculated for each. To assess the incremental value of the biomarker over established clinical variables, head-to-head comparisons of AUCs from correlated models were performed using the DeLong test. To account for the timing of cognitive decline, a survival analysis using a Cox proportional hazards regression model was performed, and the median time to cognitive impairment was calculated for the PSCI group. To assess biological compatibility with Alzheimer’s Disease (AD) pathology, participants were stratified into three risk tiers based on thresholds recently established for the Simoa ALZpath assay: Low-Risk (<0.229 pg/mL), Intermediate/Gray zone (0.229–0.516 pg/mL), and High-Risk/AD-compatible (>0.516 pg/mL) (Giacomucci et al., 2025). A *p*-value < 0.05 was considered statistically significant. All analyses were performed using SPSS 25.0 (IBM, Corp., Armonk, NY, USA) and R software (v4.4.1), and graphs were drawn using GraphPad Prism version 6 (GraphPad Software, La Jolla, CA, USA).

All procedures performed in studies involving human participants were in accordance with the ethical standards of the institutional and/or national research committee (clinical research ethics committee of Navarre; Pyto 2015/21) and with the 1964 Helsinki declaration and its later amendments or comparable ethical standards.

## 3 Results

During the follow-up period, 48 patients developed incident PSCI and were classified as PSCI-cases and 48 age and sex-matched patients without cognitive decline were selected as non-PSCI controls.

Main baseline characteristics of subjects are shown in Table 1. There were no significant differences in age, sex, stroke severity assessed by the NIHSS scale, or blood laboratory parameters between PSCI-cases and controls (all *p*-values > 0.05). No differences were observed for vascular risk factors, except for atrial fibrillation, which was more prevalent among PSCI cases (*p-*value < 0.05).

**TABLE 1:** Main features of patients with acute stroke at baseline.

Clinical features	Non-PSCI (*n* = 48)	PSCI (*n* = 48)	*p*-value
Demographic data, vascular risk factors and comorbidities			
Age, years	75 (69–81)	79 (72–86)	0.101
Sex (female)	22 (46)	22 (46)	1.000
Time to cognitive impairment (months)		19 (5–44)	
Hypertension	32 (67)	29 (60)	0.525
Diabetes mellitus	14 (29)	15 (31)	0.824
Dyslipidaemia	26 (54)	26 (54)	1.000
Never smoker	34 (74)	37 (80)	0.336
Alcohol	11 (24)	8 (18)	0.832
Atrial fibrillation	7 (15)	19 (40)	0.006
Prior stroke	6 (6)	12 (12)	0.117
Cardiopathy	7 (15)	11 (23)	0.296
Analytical parameters			
HbA1c (mmol/mol)	5.8 (5.5–6.1)	5.6 (5.5–6.2)	0.502
LDL (mg/dL)	105 (71–131)	90 (77–118)	0.388
Estimated glomerular filtration rate (eGFR) (CKD-EPI) mL/min/1.73 m^2^	60 (60–80)	60 (60–74)	0.073
Stroke characteristics and degree of dependence			
NIHSS at onset	4 (2–7.75)	5 (2.25–11)	0.180
mRS (discharge)	2 (1–3)	2 (1–3.75)	0.430
Oxfordshire classification			0.238
TACI	6 (10)	11 (23)	
PACI	27 (56)	28 (58)	
LACI	7 (15)	4 (8)	
POCI	9 (19)	5 (10)	
TOAST classification			0.256
Large-artery atherosclerosis	6 (13)	5 (11)	
Cardioembolism	13 (29)	23 (50)	
Small-vessel occlusion	6 (14)	3 (6)	
Undetermined etiology	19 (42)	13 (29)	
Other determined etiology	1 (2)	2 (4)	

All continuous variables are expressed as median (IQR) and all categorical variables as *n* (%) NIHSS, National Institutes of Health Stroke Scale; mRS, Modified Rankin Scale; TACI, total anterior circulation infarct; PACI, partial anterior circulation infarct; POCI, posterior circulation infarct; LACI, lacunar cerebral infarct.

**FIGURE 1:**
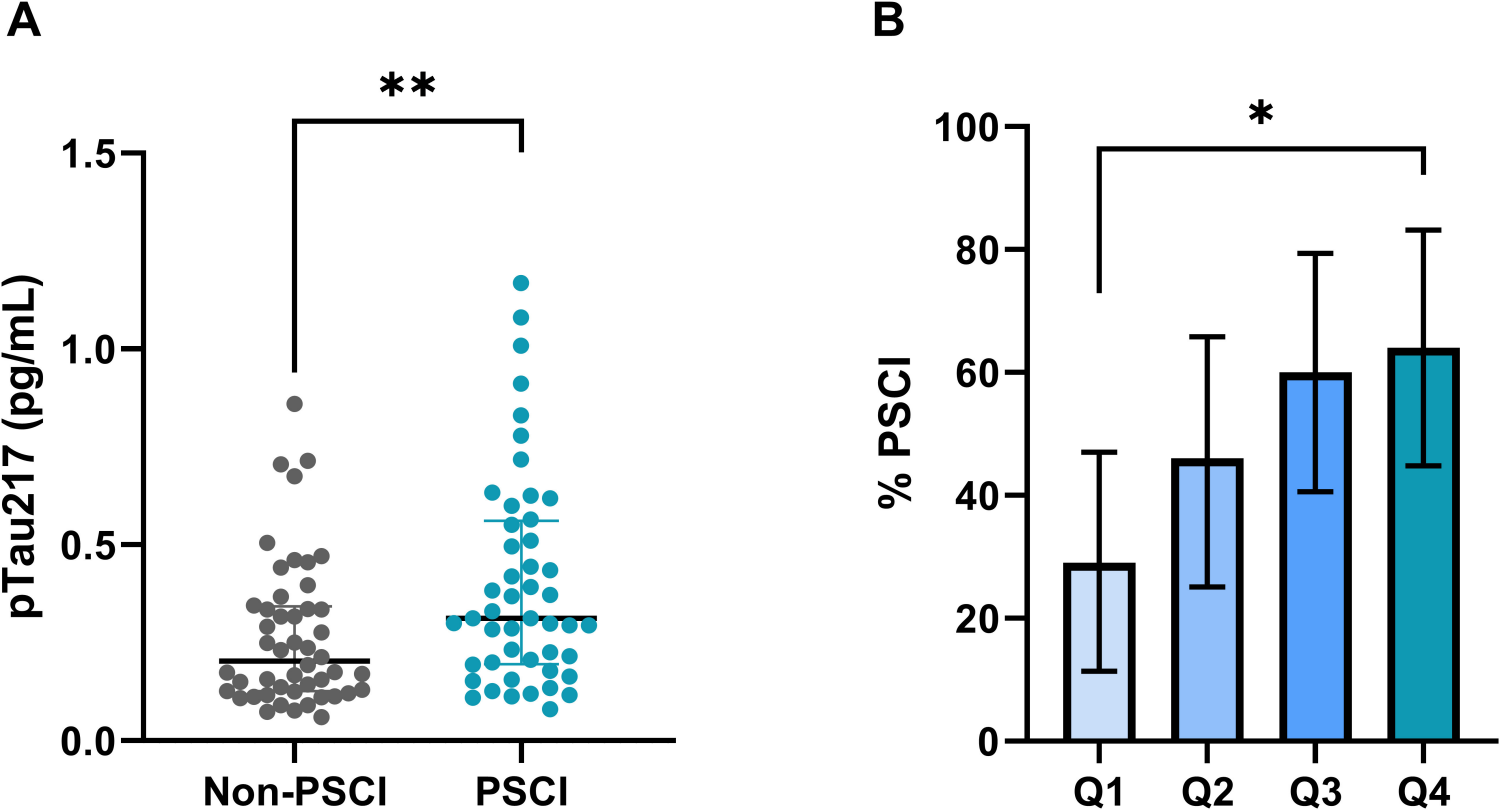
**(A)** pTau217 levels in non-PSCI controls and PSCI cases. **p-value < 0.01. **(B)** Absolute risk of PSCI development stratified by plasma pTau217 concentration quartiles. Quartile thresholds: Q1 ≤ 0.151 pg/mL (25th percentile); Q2: 0.151–0.286 pg/mL (25th–50th percentile); Q3: 0.286–0.444 pg/mL (50th–75th percentile); Q4 > 0.444 pg/mL (>75th percentile). Error bars represent 95% confidence intervals. *p* for trend = 0.017.

Regarding pTau217 measurement, all plasma samples fell within the range of detection of the assay, so they were included for statistical analysis. Plasma pTau217 levels were significantly higher in PSCI cases compared to non-PSCI controls [median (IQR) = 0.313 pg/mL (0.196–0.562) *versus* 0.203 pg/mL (0.126–0.343); *p*-value < 0.01] (Figure 1A).

No differences in pTau217 levels were observed across different stroke etiologies according to the TOAST classification. Notably, pTau217 levels showed no significant association with acute stroke severity as assessed by NIHSS scale at onset (β = 0.164, *p-*value > 0.05) nor prior stroke history (β = 0.116, *p-*value > 0.05). Furthermore, bivariate analysis revealed no significant correlation between the presence of atrial fibrillation and plasma pTau217 levels (*p-*value > 0.05), reinforcing that the biomarker’s signal is independent of stroke severity and this clinical comorbidity, respectively.

Next, we built a multivariate logistic regression model including variables showing statistical differences between PSCI cases and non-PSCI controls in the bivariate analysis to potentially improve the predictive capacity of pTau217 levels. In the model adjusted for atrial fibrillation, elevated ln-pTau217 levels were independently associated with the development of PSCI [odds ratio (OR) = 2.28; 95% CI = 1.19 – 4.37; *p*-value < 0.05] (Supplementary Table 1, Model S1). Bootstrap resampling confirmed the consistency of this association (OR = 2.47; 95% CI = 1.23 – 4.75), indicating the internal robustness of the parameter (Supplementary Table 1, Model S1). While the association remained statistically significant after adjustment, these findings are considered exploratory given the cohort size.

To provide a more stable clinical interpretation, we performed a quartile-based stratification of pTau217 levels. The absolute risk of PSCI increased progressively from 29.2% in the lowest quartile (Q1) to 64.0% in the highest quartile (Q4) (Figure 1B and Table 2). A significant linear dose-response relationship was confirmed across quartiles (OR per quartile increase = 1.57; 95% CI = 1.08–2.27; *p*-value for trend = 0.017) as shown in Table 2, reinforcing the consistency of the biomarker as a risk stratification tool and highlighting its incremental value.

**TABLE 2:** Risk stratification and logistic regression models based on pTau217 quartiles.

pTau217 categories	PSCI, *n* (%)	B	Wald	*p*-value	OR	95% CI
Trend (per quartile)	–	0.452	5.691	0.017*	1.571	1.084–2.276
Quartile 1 (Reference: 0.151 pg/mL)	7 (29)	–	–	–	1.000	–
Quartile 2 (0.151–0.286 pg/mL)	10 (46)	0.511	0.746	0.388	1.667	0.523–5.314
Quartile 3 (0.286–0.444 pg/mL)	15 (60)	1.030	3.136	0.077	2.800	0.896–8.752
Quartile 4 (>0.444 pg/mL)	16 (64)	1.322	4.875	0.027*	3.750	1.160–12.122

B, regression coefficient; Wald, Wald chi-square statistic; OR, odds ratio; CI, confidence interval. Model 1 treats quartiles as a continuous variable to test for a linear trend (per unit increase). Model 2 treats quartiles as categorical variables using the first quartile as the reference.

*p-value < 0.05.

Moreover, a progressive increase in PSCI risk was observed across increasing biomarker quartiles. As shown in Table 2, the categorical comparison revealed that patients in the highest quartile (Q4) had a significantly increased risk of developing PSCI compared to those in the first quartile (OR = 3.75; 95% CI = 1.160–12.122; *p*-value < 0.05).

To further explore the association between pTau217 and the timing of cognitive decline, we employed a survival analysis framework. The Cox proportional hazards model yielded a Hazard Ratio (HR) = 2.054 (95% CI = 0.676 – 6.240; *p*-value = 0.204) for pTau217 levels. While the direction of the estimate is consistent with the primary logistic regression findings, the association did not reach statistical significance. This result should be interpreted cautiously, as the limited sample size and low number of events substantially reduce statistical power in Cox regression analysis.

Following the risk stratification framework, 18.8% of the cohort (18/96) was classified in the high-risk/AD-compatible zone (>0.516 pg/mL). Within this group, 27.1% of PSCI cases (13/48) were biologically compatible with high AD-type risk, compared to only 10.4% of controls (5/48). Notably, 72.2% of individuals in this high-risk category eventually developed clinically documented cognitive impairment during follow-up. Conversely, over half of the controls (52.1%) fell into the low-risk category (<0.229 pg/mL), whereas only 33.3% of cases were classified as low risk. This three-tier risk stratification revealed a significant linear-by-linear association between pTau217 categories and PSCI development (*p*-value = 0.021), reflecting a progressive increase in the likelihood of cognitive decline across the low, intermediate, and high-risk groups.

Individually, pTau217 demonstrated a significant discriminative capacity (AUC = 0.660; 95% CI = 0.552–0.768; *p*-value < 0.01). To further explore the predictive utility of the biomarker in the clinical context, we constructed additional clinical reference models incorporating covariates strictly associated with clinical outcomes. The discriminative performance of pTau217 was numerically superior to both the discriminative capacity of atrial fibrillation alone (AUC = 0.625; 95% CI = 0.513–0.737; *p-*value < 0.05) and a clinical reference model incorporating age, baseline NIHSS, and atrial fibrillation (AUC = 0.657; 95% CI = 0.546–0.768; *p*-value < 0.01). The highest predictive accuracy was achieved by the integrated model combining pTau217 and atrial fibrillation, and pTau217 and the clinical reference model (AUC = 0.694; 95% CI = 0.589–0.800; *p*-value < 0.01), for both models.

Finally, we conducted a head-to-head comparison of discriminative capacities using the DeLong test to determine if the model including the biomarker provided superior predictive power over standard clinical indicators. Nevertheless, the DeLong test showed no significant differences between the models (all *p*-values > 0.05) (Supplementary Figure 1). These findings suggest that while pTau217 is an independent signal of risk, its discriminative value over models already including clinical predictors remains limited within this specific cohort.

Women typically suffer ischemic stroke at an older age than men, and advanced age is a known risk factor for cognitive decline (Guo et al., 2024). In addition, two-thirds of AD patients are women (Alzheimers Dementia, 2024). After sex stratification, we observed that pTau217 levels remained significantly higher only in females (*n* = 44) [median (IQR) PSCI cases = 0.307 pg/mL (0.173–0.582) *versus* non-PSCI controls = 0.194 pg/mL (0.119–0.338); *p*-value < 0.05], whereas these differences were no longer observed for males (*n* = 50) [median (IQR) PSCI cases = 0.322 pg/mL (0.205–0.563) *versus* non-PSCI controls = 0.221 pg/mL (0.142–0.416); *p*-value = 0.143].

## 4 Discussion

In this study, we observed an association between plasma pTau217 levels, measured during the acute phase of ischemic stroke and clinically documented PSCI within a 5-year follow-up. Our findings suggest that pTau217 may represent an early marker associated with increased risk of later cognitive impairment after stroke.

In recent years, there has been growing interest in identifying plasma biomarkers for PSCI, given the limitations of standard clinical and neuroimaging tools in predicting cognitive outcomes after stroke. For example, elevated levels of neurofilament light chain (NfL), a marker of axonal injury, have been associated with worse cognitive outcomes in stroke survivors (Marklund et al., 2021). Similarly, a reduction in plasma Aβ42 levels at 3 months post-stroke was observed in patients who developed PSCI at 1 year (Chi et al., 2019). Among tau biomarkers, pTau181 has been extensively studied in neurodegenerative diseases (Cano et al., 2024) and stroke-related cognitive impairment, though its results have not been consistent. Notably, elevated pTau181 levels in the acute phase of stroke were paradoxically associated with a decreased risk of PSCI, independent of traditional risk factors (Huang et al., 2022). Beyond these markers, glial fibrillary acidic protein (GFAP) has emerged as a significant predictor of post-stroke outcomes, reflecting astrocytic damage and neuroinflammation in the acute phase (Shan et al., 2025). However, while NfL and GFAP primarily capture the severity of the acute axonal and glial injury, their specificity for underlying neurodegenerative processes is lower. Of late, pTau217 has shown higher specificity compared to pTau181, emerging as a more precise marker of amyloid and tau pathology in AD (Sarto et al., 2025). In the context of PSCI, this distinction may be particularly relevant, as the inconsistent findings reported for pTau181 (Huang et al., 2022) suggest limited specificity for stroke-related neurodegeneration. While NfL and GFAP might reflect the weight of the vascular event, pTau217 may thus help to identify a pre-existing neurobiological vulnerability that predisposes patients to cognitive decline, regardless of stroke severity and may help to stratify the risk of PSCI development, as suggested by the 72.2% conversion rate observed in individuals in the AD-compatible zone.

The relationship between PSCI and AD is complex and multifaceted. Both conditions share common pathological mechanisms, including tau hyperphosphorylation, amyloid deposition, and neuroinflammation (Akinyemi et al., 2017; Godefroy et al., 2025b; Shi et al., 2019). Ischemic stroke can exacerbate pre-existing Alzheimer’s pathology or trigger neurodegenerative processes in susceptible individuals, leading to accelerated cognitive decline (Nucera and Hachinski, 2018; Pluta et al., 2021). This overlap underscores the importance of biomarkers like pTau217, which reflect underlying neurodegenerative changes and may help identify stroke patients at risk of developing cognitive impairment. In this cohort, pTau217 levels were not significantly associated with acute stroke severity and cumulative vascular burden. This is a crucial finding, as it may suggest that the biomarker does not merely reflect the extent of the acute brain injury, but rather may represent a baseline neurobiological vulnerability to cognitive deterioration. Our findings, showing that more than a quarter of PSCI cases exhibit pTau217 levels compatible with high AD-pathology risk according to established thresholds (Giacomucci et al., 2025), suggests that the biomarker identifies a subgroup with underlying mixed vascular-neurodegenerative copathology. In these susceptible individuals, the ischemic event likely acts as a trigger that unmasks subclinical AD-type pathology, leading to accelerated cognitive decline.

A key observation in our study is the dose-response relationship revealed through quartile analysis. Although the continuous model yielded wide confidence intervals, reflecting some model instability related to sample size and low number of events, the risk of PSCI increased progressively from 29.2% in the lowest quartile to 64.0% in the highest, with a significant linear trend. This pattern may support a potential role of pTau217 in risk stratification, although these findings should be interpreted cautiously given the exploratory nature of the study. When evaluating discriminative capacity, the model including pTau217 did not significantly outperform clinical models including age, NIHSS or atrial fibrillation according to the DeLong analysis. This underscores that its incremental value over potent clinical predictors like atrial fibrillation [which carries up to a 2.7-fold risk for cognitive decline (Koh et al., 2022)] remains modest in the acute phase. However, the consistency of the biological gradient observed across quartiles supports its interest as a complementary exploratory signal for identifying high-risk individuals. Moreover, atrial fibrillation was independently associated with PSCI, in line with previous reports (Sachdev et al., 2025), reinforcing the representativeness and validity of our cohort.

Several studies have examined sex differences in PSCI although findings have not been entirely consistent, with some studies showing mixed results or no significant differences (Dong et al., 2020; Exalto et al., 2023). In our cohort, exploratory sex-stratified analyses suggested that the observed association between pTau217 and PSCI may have been more pronounced in women. Given the limited sample size and reduced statistical power of subgroup analyses, these findings should be interpreted cautiously. Nevertheless, considering known sex-related differences in both stroke outcomes and tau-related neurobiology, further investigation in larger prospective cohorts appears warranted.

The early detection of PSCI using plasma biomarkers could have profound implications for patient care (Ma et al., 2024). Identifying high-risk individuals during the acute phase of stroke could enable the implementation of targeted interventions, such as closer and more personalized clinical follow-up, cognitive stimulation therapy, or lifestyle modifications, to prevent or delay cognitive decline. Moreover, combining biomarkers with neuroimaging and clinical data could enhance risk stratification and enable personalized treatment strategies. For example, patients with elevated pTau217 levels could be prioritized for more intensive follow-up or enrolled in clinical trials of disease-modifying therapies.

While our findings are promising, several limitations should be acknowledged. First, the sample size was relatively small, and the study was conducted at a single center, which may limit the generalizability of the results. In addition, the limited number of events may have contributed to model instability, wide confidence intervals, and potential overfitting or sparse-data bias, despite the consistency observed in bootstrapping analysis, and precluded definitive conclusions from the complementary survival analysis. Secondly, the diagnosis was based on cognitive dysfunction documented in the medical record at any point during the 5-year follow-up period. Therefore, the study should be interpreted as evaluating clinically documented cognitive impairment rather than prospectively adjudicated PSCI using standardized cognitive scales. Furthermore, detailed and standardized information regarding prior cerebrovascular burden, including silent infarcts or leukoaraiosis, or cognitive reserve, including educational level, was not systematically available for all patients, which may have introduced residual confounding related to baseline cognitive reserve and vascular brain injury and may limit the interpretation of pTau217 as an independent marker of cognitive vulnerability. Thirdly, we did not assess other biomarkers, such as Aβ42,NfL or pTau181, which could provide additional insights into the pathophysiology of PSCI and help clarify the incremental value of pTau217 relative to other neurodegenerative biomarkers. Future studies should explore the integration of multiple plasma biomarkers, neuroimaging markers and clinical variables to improve risk stratification and better elucidate the mechanisms linking stroke to cognitive impairment.

In conclusion, plasma pTau217 levels measured during the acute phase of ischemic stroke may help to assess long-term cognitive risk after stroke. Our findings suggest that plasma pTau217 may be associated with neurobiological vulnerability to cognitive impairment after stroke, potentially reflecting processes that extend beyond acute vascular injury alone. Further research is needed to validate these results in larger, more diverse cohorts, to assess the potential sex dimorphism and to explore the integration of pTau217 with other biomarkers and clinical data.

## Data Availability

The original contributions presented in this study are included in the article/Supplementary material, further inquiries can be directed to the corresponding authors.

## References

[B1] Adams H. Bendixen B. Kappelle L. Biller J. Love B. Gordon D. et al. (1993). Classification of subtype of acute ischemic stroke. Definitions for use in a multicenter clinical trial. TOAST. Trial of Org 10172 in Acute *Stroke* Treatment. *Stroke* 24 35–41. 10.1161/01.str.24.1.35 7678184

[B2] Akinyemi R. O. Allan L. Oakley A. Kalaria R. (2017). Hippocampal neurodegenerative pathology in post-stroke dementia compared to other dementias and aging controls. *Front Neurosci.* 11:717. 10.3389/fnins.2017.00717 29311794 PMC5742173

[B3] Alzheimers Dementia. (2024). 2024 Alzheimer’s disease facts and figures. *Alzheimers Dement*. 20 3708–3821. 10.1002/alz.13809 38689398 PMC11095490

[B4] Bamford J. Sandercock P. Dennis M. Burn J. Warlow C. (1991). Classification and natural history of clinically identifiable subtypes of cerebral infarction. *Lancet* 337 1521–1526. 10.1016/0140-6736(91)93206-o 1675378

[B5] Benussi A. Huber H. Tan K. Cantoni V. Rivolta J. Cotelli M. et al. (2025). Plasma p-tau217 and neurofilament/p-tau217 ratio in differentiating Alzheimer’s disease from syndromes associated with frontotemporal lobar degeneration. *Alzheimers Dement*. 21:e14482. 10.1002/alz.14482 39776166 PMC11848195

[B6] Cano A. Capdevila M. Puerta R. Arranz J. Montrreal L. Rojas I. et al. (2024). Clinical value of plasma pTau181 to predict Alzheimer’s disease pathology in a large real-world cohort of a memory clinic. *EBioMedicine* 108:105345. 10.1016/j.ebiom.2024.105345 39299003 PMC11424964

[B7] Chi N. Chao S. Huang L. Chan L. Chen Y. Chiou H. et al. (2019). Plasma amyloid beta and tau levels are predictors of post-stroke cognitive impairment: A longitudinal study. *Front. Neurol.* 10:715. 10.3389/fneur.2019.00715 31312178 PMC6614443

[B8] Cui P. Ma X. Li H. Lang W. Hao J. (2018). Shared biological pathways between Alzheimer’s disease and ischemic stroke. *Front. Neurosci*. 12:605. 10.3389/fnins.2018.00605 30245614 PMC6137293

[B9] Dong L. Briceno E. Morgenstern L. Lisabeth L. (2020). Poststroke cognitive outcomes: Sex differences and contributing factors. *J. Am. Heart Assoc.* 9:e016683. 10.1161/JAHA.120.016683 32633589 PMC7660722

[B10] Dong S. Maniar S. Manole M. Sun D. (2018). Cerebral hypoperfusion and other shared brain pathologies in ischemic stroke and Alzheimer’s disease. *Transl. Stroke Res.* 9 238–250. 10.1007/s12975-017-0570-2 28971348 PMC9732865

[B11] El Husseini N. Katzan I. Rost N. Blake M. Byun E. Pendlebury S. et al. (2023). Cognitive impairment after ischemic and hemorrhagic *stroke*: A scientific statement from the American Heart Association/American Stroke Association. *Stroke* 54 e272–e291. 10.1161/STR.0000000000000430 37125534 PMC12723706

[B12] Exalto L. Weaver N. Kuijf H. Aben H. Bae H. Best J. et al. (2023). Sex differences in poststroke cognitive impairment: A multicenter study in 2343 patients with acute ischemic *stroke*. *Stroke* 54 2296–2303. 10.1161/STROKEAHA.123.042507 37551589 PMC10453354

[B13] GBD 2019 Stroke Collaborators. (2021). Global, regional, and national burden of stroke and its risk factors, 1990-2019: A systematic analysis for the Global burden of disease study 2019. *Lancet Neurol.* 20 795–820. 10.1016/S1474-4422(21)00252-0 34487721 PMC8443449

[B14] Giacomucci G. Crucitti C. Ingannato A. Moschini V. Bagnoli S. Pozzi F. et al. (2025). The two cut-offs approach for plasma p-tau217 in detecting Alzheimer’s disease in subjective cognitive decline and mild cognitive impairment. *Alzheimers Dement.* 17:e70116. 10.1002/dad2.70116 40357140 PMC12066393

[B15] Godefroy O. Aarabi A. Béjot Y. Biessels G. Glize B. Mok V. et al. (2025a). Are we ready to cure post-stroke cognitive impairment? Many key prerequisites can be achieved quickly and easily. *Eur. Stroke J.* 10 22–35. 10.1177/23969873241271651 39129252 PMC11569528

[B16] Godefroy O. Trinchard N. Marchal E. Lamy C. Canaple S. Meyer M. et al. (2025b). Do amyloid cerebral deposits influence the long-term poststroke cognitive outcome?: The IDEA3 study. *Stroke* 56 74–83. 10.1161/STROKEAHA.124.049147 39648884

[B17] Guo X. Phan C. Batarseh S. Wei M. Dye J. (2024). Risk factors and predictive markers of post-stroke cognitive decline-A mini review. *Front. Aging Neurosci.* 16:1359792. 10.3389/fnagi.2024.1359792 38414631 PMC10896992

[B18] Huang L. Chao S. Hu C. Chien L. Chiou H. Lo Y. et al. (2022). Plasma Phosphorylated-tau181 is a predictor of post-stroke cognitive impairment: A longitudinal study. *Front. Aging Neurosci.* 14:889101. 10.3389/fnagi.2022.889101 35572134 PMC9099290

[B19] Koh Y. H. Lew L. Franke K. Elliott A. Lau D. Thiyagarajah A. et al. (2022). Predictive role of atrial fibrillation in cognitive decline: A systematic review and meta-analysis of 2.8 million individuals. *Europace* 24 1229–1239. 10.1093/europace/euac003 35061884 PMC9435641

[B20] Ma Y. Chen Y. Yang T. He X. Yang Y. Chen J. et al. (2024). Blood biomarkers for post-stroke cognitive impairment: A systematic review and meta-analysis. *J. Stroke Cerebrovasc. Dis.* 33:107632. 10.1016/j.jstrokecerebrovasdis.2024.107632 38417566

[B21] Marklund N. Vedung F. Lubberink M. Tegner Y. Johansson J. Blennow K. et al. (2021). Tau aggregation and increased neuroinflammation in athletes after sports-related concussions and in traumatic brain injury patients - A PET/MR study. *Neuroimage Clin*. 30:102665. 10.1016/j.nicl.2021.102665 33894460 PMC8091173

[B22] NIH. (2025). NIH Stroke Scale | National Institute of Neurological Disorders and Stroke. Bethesda, MD: National Institute of Neurological Disorders and Stroke (NINDS).

[B23] Nucera A. Hachinski V. (2018). Cerebrovascular and Alzheimer disease: Fellow travelers or partners in crime? *J. Neurochem.* 144 513–516. 10.1111/jnc.14283 29266273

[B24] Palmqvist S. Janelidze S. Quiroz Y. Zetterberg H. Lopera F. Stomrud E. et al. (2020). Discriminative accuracy of plasma Phospho-tau217 for Alzheimer Disease vs other neurodegenerative disorders. *JAMA* 324 772–781. 10.1001/jama.2020.12134 32722745 PMC7388060

[B25] Pluta R. Januszewski S. Czuczwar S. (2021). Brain ischemia as a prelude to Alzheimer’s disease. *Front. Aging Neurosci.* 13:636653. 10.3389/fnagi.2021.636653 33679381 PMC7931451

[B26] Rawat P. Sehar U. Bisht J. Selman A. Culberson J. Reddy P. (2022). Phosphorylated Tau in Alzheimer’s disease and other tauopathies. *Int. J. Mol. Sci.* 23:12841. 10.3390/ijms232112841 36361631 PMC9654278

[B27] Rissin D. Kan C. Campbell T. Howes S. Fournier D. Song L. et al. (2010). Single-molecule enzyme-linked immunosorbent assay detects serum proteins at subfemtomolar concentrations. *Nat. Biotechnol.* 28 595–599. 10.1038/nbt.1641 20495550 PMC2919230

[B28] Rost N. Brodtmann A. Pase M. van Veluw S. Biffi A. Duering M. et al. (2022). Post-stroke cognitive impairment and dementia. *Circ. Res.* 130 1252–1271. 10.1161/CIRCRESAHA.122.319951 35420911

[B29] Rost N. Meschia J. Gottesman R. Wruck L. Helmer K. Greenberg S. (2021). Cognitive impairment and dementia after *stroke*: Design and rationale for the discovery study. *Stroke* 52 e499–e516. 10.1161/STROKEAHA.120.031611 34039035 PMC8316324

[B30] Sachdev P. Bentvelzen A. Kochan N. Jiang J. Hosoki S. Koncz R. et al. (2025). Revised diagnostic criteria for vascular cognitive impairment and dementia-the VasCog-2-WSO criteria. *JAMA Neurol*. 82 1103–1112. 10.1001/jamaneurol.2025.3242 40955506 PMC12441927

[B31] Sarto J. Esteller-Gauxax D. Guillén N. Falgàs N. Borrego-Écija S. Massons M. et al. (2025). Accuracy and clinical applicability of plasma tau 181 and 217 for Alzheimer’s disease diagnosis in a memory clinic cohort. *J. Neurol.* 272:160. 10.1007/s00415-025-12897-5 39849125

[B32] Sexton E. McLoughlin A. Williams D. Merriman N. Donnelly N. Rohde D. et al. (2019). Systematic review and meta-analysis of the prevalence of cognitive impairment no dementia in the first year post-stroke. *Eur. Stroke J.* 4 160–171. 10.1177/2396987318825484 31259264 PMC6591758

[B33] Shan W. Jiang R. Wang J. Xu G. Zhao J. Zhai G. et al. (2025). High serum glial fibrillary acidic protein levels are associated with increased risk of post-stroke cognitive impairment. *Front. Aging Neurosci.* 17:1546270. 10.3389/fnagi.2025.1546270 40421099 PMC12104187

[B34] Shi E. Shi K. Qiu S. Sheth K. Lawton M. Ducruet A. (2019). Chronic inflammation, cognitive impairment, and distal brain region alteration following intracerebral hemorrhage. *FASEB J*. 33 9616–9626. 10.1096/fj.201900257R 31145859

[B35] Xia Y. Prokop S. Giasson B. I. (2021). “Don’t Phos Over Tau”: Recent developments in clinical biomarkers and therapies targeting tau phosphorylation in Alzheimer’s disease and other tauopathies. *Mol. Neurodegener.* 16:37. 10.1186/s13024-021-00460-5 34090488 PMC8180161

[B36] Yu L. Boyle P. Janelidze S. Petyuk V. Wang T. Bennett D. et al. (2023). Plasma p-tau181 and p-tau217 in discriminating PART, AD and other key neuropathologies in older adults. *Acta Neuropathol.* 146 1–11. 10.1007/s00401-023-02570-4 37031430 PMC10261204

