# Supplementary material for: Association of acute-phase plasma pTau217 levels with long-term post-stroke cognitive impairment
Source: Front Aging Neurosci. 2026 Jul 23;18:1839662. doi: 10.3389/fnagi.2026.1839662 (PMC13442532; doi:10.3389/fnagi.2026.1839662)
Supplement: Supplementary file 1 [file Supplementary_file_1.docx]

Supplementary Material

# 1 Supplementary Figures and Tables

**Supplementary Table 1.** **Complementary logistic regression models for PSCI prediction.** This table provides detailed statistical parameters for the supporting multivariable logistic regression analyses. Model S1 evaluates the independent predictive value of log-transformed (ln) pTau217 levels when adjusted for the presence of atrial fibrillation. Internal validation was performed using bootstrap resampling (1,000 iterations). *Abbreviations: B, regression coefficient; Wald, Wald chi-square statistic; p-value, probability value; OR, odds ratio; CI, confidence interval; . *p-value <0.05.*

|  | **Variable** | **B** | **Wald** | ***p-*value** | **OR** | **95%CI** |
| --- | --- | --- | --- | --- | --- | --- |
| **Model S1** | Ln-pTau217 | 0.824 | 6.145 | 0.013* | 2.279 | 1.188– 4.372 |
|  | Ln-pTau217 (bootstrap) | - | - | - | 2.470 | 1.231 - 4.752 |
|  | Atrial fibrillation | 1.225 | 5.525 | 0.019* | 3.404 | 1.226 - 9.452 |
|  | Constant | 0.783 | 2.321 | 0.128 | 02.187 |  |

**Supplementary Figure 1.** **Comparative ROC curve analysis for PSCI prediction.** The graph represent the AUCs (95% CI) of five predictive multivariable logistic regression models: (1) ln-pTau217 alone (AUC = 0.660), (2) atrial fibrillation alone (AUC = 0.625), (3) the clinical reference model (age, NIHSS, and atrial fibrillation; AUC = 0.657), (4) an adjusted model combining ln-pTau217 and atrial fibrillation (AUC = 0.694) and (5) the comprehensive integrated model (clinical reference + ln-pTau217; AUC = 0.694). Abbreviations: AF: atrial fibrillation; AUC, area under the curve; CI, confidence interval; ln-pTau217, log-transformed pTau217; NIHSS, National Institutes of Health Stroke Scale; ROC: Receiver Operating Characteristic.
